# Intussusception caused by a small intestinal lipoma with ectopic gastric mucosa containing gastric cystica profunda component cells within the inverted Meckel’s diverticulum: a case report

**DOI:** 10.1186/s40792-020-01061-y

**Published:** 2020-11-13

**Authors:** Natsuko Yamauchi, Takashi Ito, Hiroki Matsuoka, Teruhiro Chohno, Hiroshi Hasegawa, Yoshihiro Kakeji, Takamasa Ohnishi

**Affiliations:** 1 Department of Surgery, Nishiwaki Municipal Hospital, 652-1 Shimotoda, Nishiwaki, Hyogo 677-0043 Japan; 2 Division of Gastrointestinal Surgery, Department of Surgery, Kobe University Graduate School of Medicine, 7‐5‐2 Kusunoki‐cho, Chuo‐ku, Kobe, Hyogo 650‐0017 Japan; 3 Department of Diagnostic Pathology, Nishiwaki Municipal Hospital, 652-1 Shimotoda, Nishiwaki, Hyogo 677-0043 Japan

**Keywords:** Intussusception, Ectopic gastric mucosa, Gastritis cystica profunda (GCP), Lipoma, Inverted Meckel’s diverticulum

## Abstract

**Background:**

Lipomas are the most common cause of intussusception in adults. To our knowledge, however, no cases of lipoma and ectopic gastric mucosa with gastritis cystica profunda (GCP) have been reported. We report a case of intussusception caused by a small intestinal lipoma with ectopic gastric mucosa containing GCP-component cells within the inverted Meckel’s diverticulum.

**Case presentation:**

A female in her 40s underwent computed tomography for postoperative follow-up of left breast cancer. A tumor, suspected to be a lipoma, was found in the ileum. Since there were no symptoms, the patient underwent regular follow-up. However, gradual enlargement was observed, and surgery was recommended due to the risk of intussusception. After reduction via the Hutchinson technique, laparoscopically assisted partial resection of the small intestine was performed due to suspicion that the tumor was causing intussusception starting from the ileum. Histopathologic examinations revealed proliferation of mature adipose tissue in the subserosal layer, which was diagnosed as lipoma. Furthermore, adipose tissue was found in the stem area and accordingly, we diagnosed lipoma associated with the inverted Meckel’s diverticulum. Moreover, gastric mucosa-like crypt epithelium and proper glandular tissue were identified in the mucosal membrane at the area of onset, and signs of gastric pit dilatation over the submucosa and crypt epithelium hyperplasia were observed. Diagnosis was ectopic gastric mucosa containing GCP component tissue.

**Conclusions:**

Intussusception in the small intestine complicated with lipoma and ectopic gastric mucosa with GCP within the Meckel’s diverticulum has not been reported, demonstrating the rarity of our case.

## Background

Intussusception in adults is a relatively rare condition that is often caused by neoplastic disease [1]. Intussusception is a common condition in children, with > 90% of cases said to be idiopathic without any organic disease [2]. Approximately 10% of all intussusceptions have been reported in adults [3, 4]. In small intestine intussusception, benign tumors accounted for 63–71% of cases, with lipoma being the most common type [5, 6]. Furthermore, intussusception caused by the Meckel’s diverticulum is rare with 3.3–5% of all adult intussusception [7]. Moreover, to our knowledge, no reports exist of patients with gastritis cystica profunda (GCP) in the ectopic gastric mucosa within the inverted Meckel’s diverticulum.

We report on a rare case of bowel intussusception due to ectopic gastric mucosa with small bowel lipoma and GCP within inverted Meckel’s diverticulum.

## Case presentation

A female patient in her 40s presented without a chief complaint. She had undergone a partial left mastectomy for left breast cancer 2 years previously and currently was on adjuvant hormone therapy. Postoperatively, the patient was undergoing follow-up examinations at our breast oncology department. Computed tomography (CT) revealed a neoplastic lesion (lipoma) with invagination in the small intestine.

**Fig. 1:**
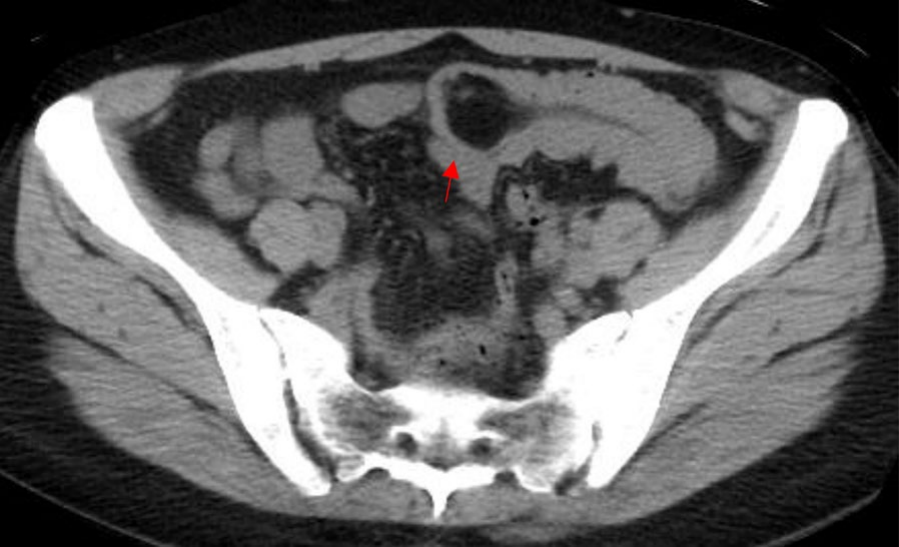
CT scans of the abdomen. A fatty mass (indicated by the red arrow), 36 mm in diameter, with a long peduncle was found in the small intestine. The pseudo-kidney sign was noted, and intussusception appeared to be present

At admission, the patient was 161.8 cm tall, weighed 60.6 kg and had a body mass index of 23.15. The abdomen was flat and soft, with no tenderness or palpable mass. Admission laboratory blood tests revealed no noteworthy findings. Plain abdominal CT showed a fatty mass in the small intestine (Fig. 1). Pseudo-kidney and target signs were observed. The tumor was 35 mm in diameter. The patient was followed, but there was no improvement in the tendency for tumor enlargement and pseudo-kidney signs. Based on these findings, surgery was recommended due to tumor enlargement and the possibility of intussusception from the tumor in the small intestine.

Laparoscopic-assisted partial resection of the small intestine was performed with the patient under general anesthesia. A pedunculated neoplastic lesion was found approximately 30 cm from the terminal end of the ileum on the oral side and intussusception was observed over approximately 20 cm on the anal side. Although edema was noted together with the duplicated intestine, there was good color tone and there were no ischemic changes. The intussusception was released via the Hutchinson technique, and partial ileal resection was performed.

**Fig. 2:**
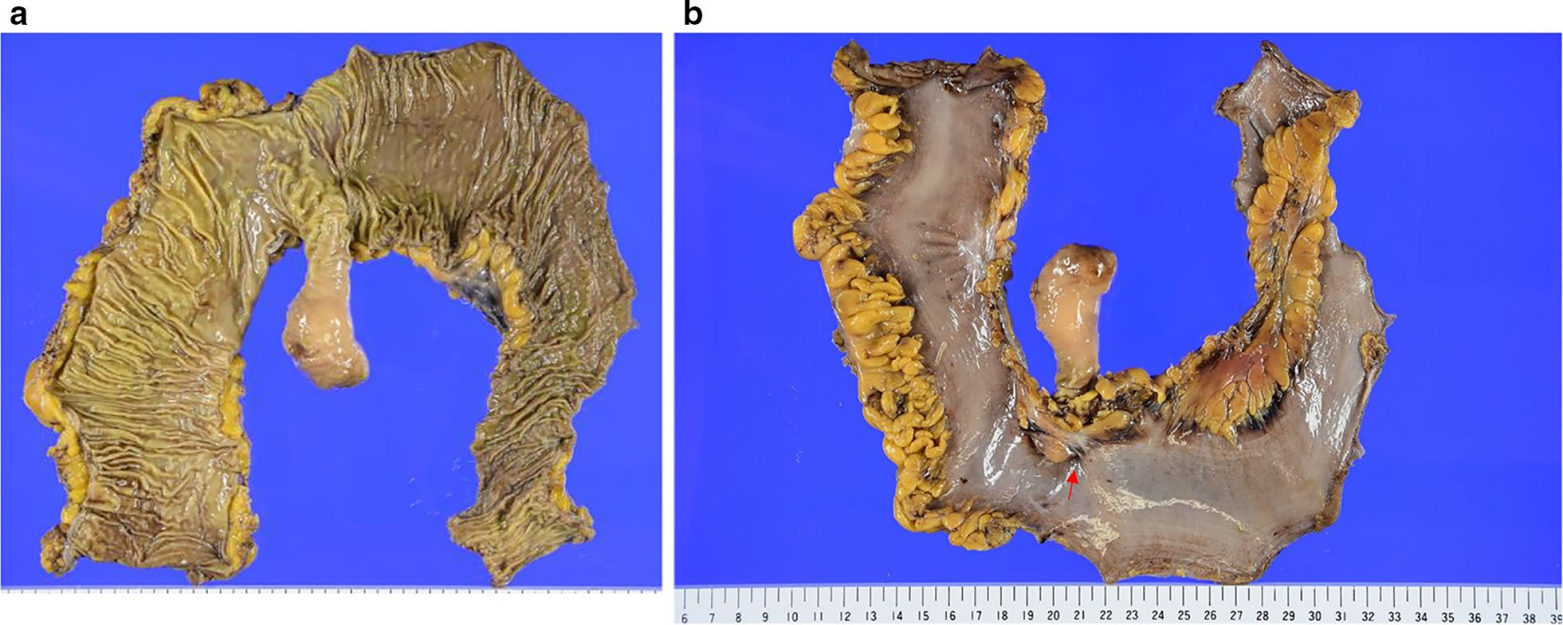
Macroscopic findings from the resected specimen. **a** A pedunculated 38-mm mass with a club-shaped apex was found in the ileum approximately 30 cm from the terminal end. **b** An excavation was observed on the serous surface (indicated by the red arrow)

The resected specimens revealed a pedunculated, club-shaped mass protruding from the mucosa into the lumen connected to the wall of the small intestine. The transverse and long axis tumor diameters were 36 and 78 mm, respectively (Fig. 2a) and an excavation was observed on the serous surface (Fig. 2b).

**Fig. 3:**
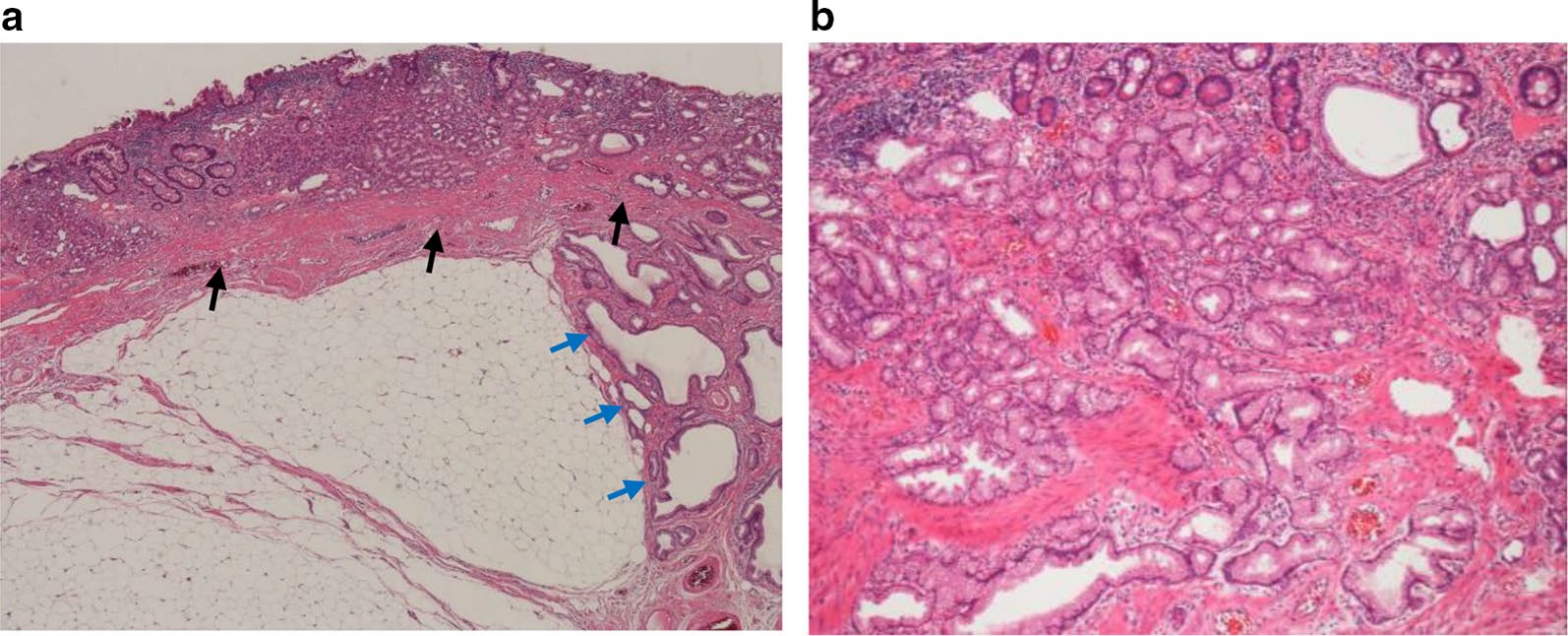
Histological findings. **a** HE (×40). Growth of mature adipose tissue was observed in the submucosa of the area of tumor onset. The muscularis mucosae (black arrow) and GCP-like glandular ducts were observed in the submucosal area (blue arrow). **b** HE (×100). A mix of gastric mucosa-like crypt epithelium and proper glandular tissue was found in the small intestine epithelium. Signs of dilatation of the deeper parts of the gastric pit over the submucosa and hyperplasia of the crypt epithelium were observed

Histopathologically, the mucosal epithelium showed inflammatory cell infiltration in the superficial layer of the area of tumor onset and the submucosa showed proliferation of atypical mature adipocytes (Fig. 3a). Gastric mucosa-like crypt epithelium and proper glandular tissue were found in the small intestine epithelium. Additionally, GCP-like glandular ducts were seen over the submucosa, and dilatation of the deep gastric pits and hyperplasia of the crypt epithelium were noted (Fig. 3b).

**Fig. 4:**
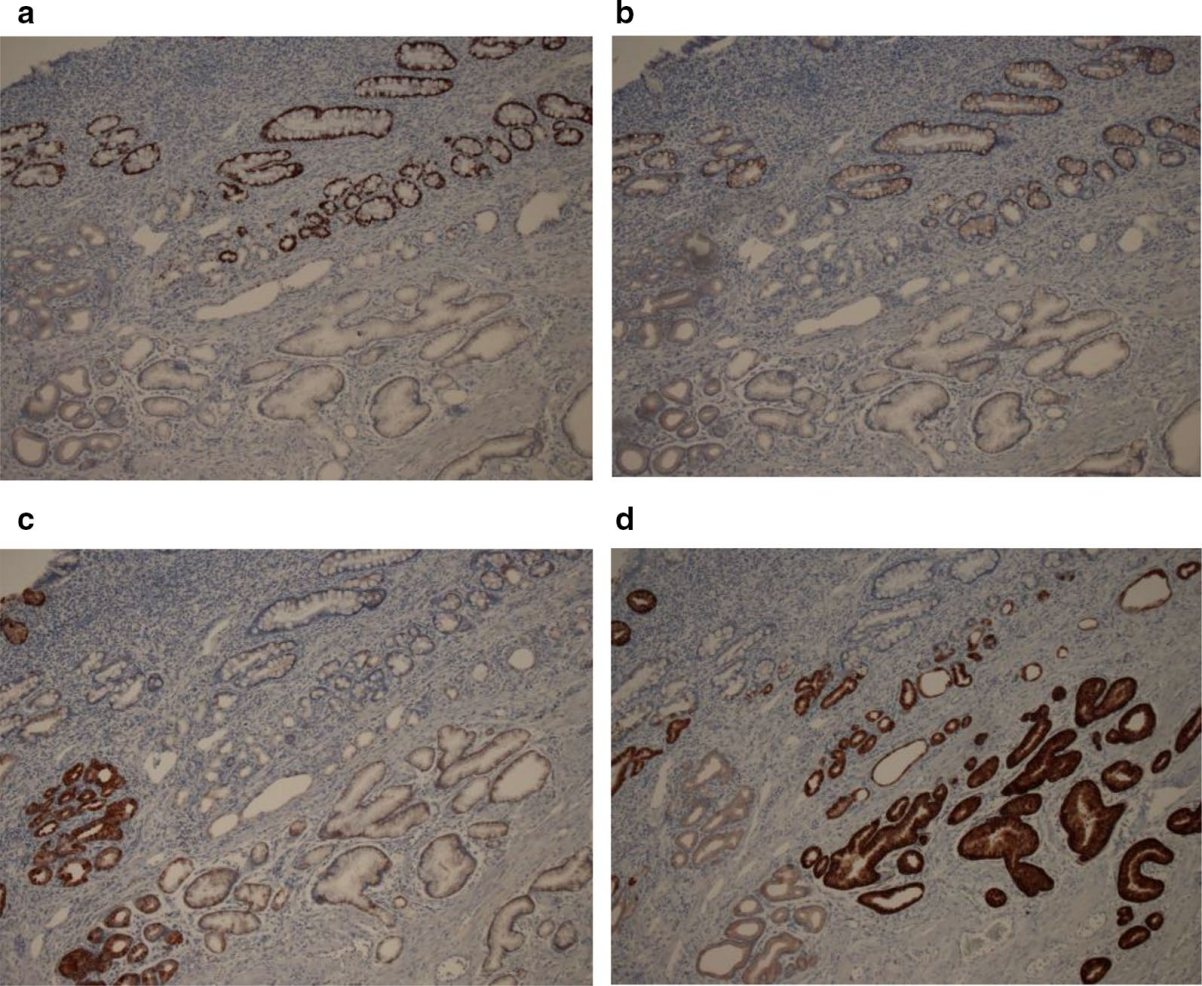
Immunohistochemistry. **a** CDX2 (×100). The mucosal epithelium of the small intestine was positive and the gastric crypt-like epithelium was negative. **b** MUC2 (×100). The goblet epithelium of the mucosa of the small intestine was positive and the gastric crypt-like epithelium was negative. **c** MUC5AC (×100). The mucosal epithelium of the small intestine was negative and the superficial gastric crypt-like epithelium was positive. **d** MUC6 (×100). The mucosal epithelium of the small intestine was negative and the gastric crypt-like epithelium was positive

Immunohistochemical findings (Fig. 4a–d) revealed that the mucosal epithelium of the small intestine was positive for CDX2, negative for MUC5AC, and negative for MUC6. The gastric crypt-like epithelium was negative for CDX2 and MUC2, and positive for MUC6. The goblet epithelium of the small intestine was positive for MUC2. The superficial gastric crypt-like epithelium and parts of the deep GCP were positive for MUC5AC. Gastrin was negative.

The patient progressed well postoperatively, and food intake began on day 4 postoperatively. The patient was discharged from the hospital on day 9.

## Discussion

We report a rare case of intussusception of the small intestine caused by lipoma with ectopic gastric mucosa including GCP within the inverted Meckel’s diverticulum.

Ectopic gastric mucosa originates from the transposition or invasive migration of gastric tissue, mutation and dysplasia of intestinal mucosa [8], and vitelline vascular origins in the fetal stage [9]. The most common sites are the entire gastrointestinal tract and biliary system; onset is possible from any primitive gut site [8]. Intussusception caused by the inverted Meckel’s diverticulum with ectopic gastric mucosa has been reported [10]. As a characteristic of the inverted Meckel’s diverticulum, the morphology of the inverted Meckel’s diverticulum may involve serosal adipose tissue; it is often observed along with a mesentery containing adipose tissue, such as one crossing the ileal wall. If the diverticulum is inverted because of this, mesenteric adipose tissue is believed to be involved in the form of a lipoma [11]. Consequently, connections to the mesenteric adipose tissue are observed. In our patient, the adipose tissue was solitary—tissues were found mainly in the submucosal layer without connections to the mesenteric adipose tissue. It has been reported that ectopic gastric mucosa of the gastrointestinal tract is associated with Meckel’s diverticulum, gastrointestinal duplication, or ectopic pancreas [12, 13]. Thus, we diagnosed ectopic gastric mucosa complicated with lipoma combined with the inverted Meckel’s diverticulum. Until now, there have been sporadic reports of the ectopic gastric mucosa in the small intestine caused by the intussusception; however, no reports on ectopic gastric mucosa with internal lipoma exist within the inverted Meckel’s diverticulum.

Polypoid mucosa in the gastrointestinal anastomoses of the remnant stomach has been reported as GCP [14]; pathological features include hyperplasic gastric pit epithelium, atrophy of corpus glands, hyperplasic pseudopyloric glands, and cystic dilatation; cysts are observed in the lamina propria and submucosa. However, a report on lesion onset at the gastrojejunostomy site after gastrectomy suggests GCP [15]. Cysts with histologic features of GCP but confined to the lamina propria were distinguished from those invading from the muscularis mucosae to the submucosa, and the latter were proposed to be GCP. GCP are found in gastric remnants and caused by suturing and inflammation due to ischemia or reflux of duodenal fluid [14, 15]. Similar conditions in nonresected stomachs have been considered as GCP. Studies on heterozygous TGF-β1 knockout mice have shown GCP-like lesion onset [16], but a definite etiology is unknown.

Here, gastric crypt-like epithelium and dilated cystic glandular duct structures were observed in the lamina propria and submucosa. G cells did not produce gastrin in the pyloric antrum of the stomach, but CDX2 was expressed in epithelial cell nuclei in the intestine from the duodenum to the rectum [17]. Mucins are classified as secreted gel-forming mucins (MUC2, MUC5AC, and MUC6), transmembrane mucins (MUC1), and others that are uncategorized [18]. MUC1 is expressed on the apical surfaces of epithelial cells, MUC2 is a marker of intestinal phenotypic traits of goblet cells in the mucosa of both intestines becoming positive, and MUC5AC is expressed in gastric crypt epithelial cells [19].

MUC6 is expressed in gastric pyloric and cardiac glands, gastric accessory cells, duodenal Brunner glands, and esophageal cardiac glands [20]. CDX2, MUC2, MUC5AC, and MUC6 confirmed the ectopic gastric crypt epithelium. This is an extremely rare case because GCP identification in ectopic gastric mucosa has not been reported. GCP is closely related to cancer; due to the high proliferative potential of GCP-component cells, they present along with cancer and some may be precancerous lesions [21, 22]. However, the pathogenesis is unknown. Thus, possible carcinogenesis was not ruled out; however, we consider it highly significant that we could resect the lesion.

## Conclusions

We reported on an extremely rare case of a patient with intussusception caused by a small intestinal lipoma accompanied by ectopic gastric mucosa containing GCP within the inverted Meckel’s diverticulum.

## Data Availability

All data generated or analyzed during this study are included in this published article.

## Abbreviations

GCP: Gastritis cystica profunda
CT: Computed tomography
